# Medical device assessment: scientific evidence examined by the French national agency for health – a descriptive study

**DOI:** 10.1186/1471-2458-12-585

**Published:** 2012-08-01

**Authors:** Laure Huot, Evelyne Decullier, Karen Maes-Beny, Francois R Chapuis

**Affiliations:** 1 Hospices Civils de Lyon, Pôle Information Médicale Evaluation Recherche, Unité de Recherche Clinique, Lyon, 69003, France; 2 Université de Lyon, RECIF, EA Santé Individu Société 4129, Lyon, 69003, France; 3 Université Lyon 1, Lyon, 69003, France; 4 Conférence Nationale des Comités de Protection des Personnes dans la recherche biomédicale, Comité de Protection des Personnes Sud-Est III, Lyon, 69003, France

**Keywords:** Implantable medical devices, Health technology assessment, Level of evidence, Clinical trials

## Abstract

**Background:**

Scientific evidence supports decision-making on the use of implantable medical devices (IMDs) in clinical practice, but IMDs are thought to be far less investigated than drugs. In the USA, studies have shown that approval process of high-risk medical devices was often based on insufficiently robust studies, suggesting that evidence prior to marketing may not be adequate. This study aimed to ascertain level of evidence available for IMDs access to reimbursement in France.

**Methods:**

The objective was to examine the scientific evidence used for IMDs assessment by the French National Authority for Health. We collected all public documents summarising supportive clinical data and opinions concerning IMDs issued in 2008. An opinion qualifies the expected benefit (EB) of the IMD assessed as sufficient or insufficient, and if sufficient, the level of improvement of the expected benefit (IEB) on a scale from major (level I) to no improvement (level V). For each opinion, the study with the highest level of evidence of efficacy data, and its design were collected, or, where no studies were available, any other data sources used to establish the opinion.

**Results:**

One hundred and two opinions were analysed, with 72 reporting at least one study used for assessment (70.6%). When considering the study with the highest level of evidence: 34 were clinical non-comparative studies (47.2%); 29 were clinical comparative studies of which 25 randomised controlled trials (40.3%); 5 were meta-analyses of randomised controlled trials (6.9%); and 4 were systematic literature reviews (5.6%). The opinions were significantly different according to the study design (p < 0.001). The most frequent design for insufficient EB, IEB level V and IEB level IV was a non-comparative study (10/19, 52.6%; 15/24, 62.5%; and 8/15, 53.3%; respectively). For the 30 opinions with no supporting clinical study, 16 (53.3%) were based on an expert-based process, 9 (30.0%) were based on the conclusions of a previous opinion (all concluding IEB level V), and 5 (16.7%) reported no data (concluding insufficient EB for 4 and IEB level V for 1).

**Conclusions:**

This study confirmed that level of evidence of clinical evaluation of IMDs is low and needs to be improved.

## Background

By the late 1990s, the US Food and Drug Administration (FDA) had given marketing approval to around 500,000 medical devices, produced by approximately 23,000 different manufacturers, and it has been estimated that 4% of the US population has at least one implanted medical device [1,2]. The US medical device market was estimated in 2011 at US$105.8 billion. In Europe, there are almost 22,500 medical technology firms, which generate annual sales of €95 billion [3]. In 2006, the medical device market in France comprised €19 billion in sales, representing 12% of medical and health care consumption [4].

Most new medical technologies are intended to improve quality of care but they also increase health spending [5]. For this reason Health Technology Assessment (HTA), which is defined as the systematic evaluation of the properties, effects, and/or impacts of healthcare technology [6], has been developed to inform healthcare providers, payers and patients about technology-related clinical and cost effectiveness. In France, the government set up the French National Authority for Health (*Haute Autorité de Santé,* HAS) in August 2004, whose activities are designed to improve the quality of patient care and guarantee equity within the healthcare system. These activities include the assessment of medical devices based on scientific expertise, carried out by a dedicated committee (*Commission Nationale d’Evaluation des Dispositifs Médicaux et Technologies de Santé,* CNEDIMTS) [7]. The aim of these assessments is to provide “opinions” to health authorities that contain the information needed to support decisions regarding the reimbursement. All assessed medical devices qualified with “sufficient expected clinical benefit” are included on the list of products and services qualifying for reimbursement [8].

In this context, a manufacturer who seeks reimbursement by the French Health Insurance system for the cost of a medical device has two options. If the medical device responds to a generic definition already included on the list qualifying for reimbursement, it can be registered directly. In case there is no generic definition for the medical device, the manufacturer has to apply for specific CNEDIMTS’ assessment (brand name registration), and to submit a dossier containing clinical data. These data should demonstrate the value of the medical device, i.e. what is the expected clinical benefit and whether it is sufficient to justify its inclusion on the list qualifying for reimbursement, and should propose what improvement in terms of clinical benefit may be expected in comparison with the existing standard of care. The CNEDIMTS’ members base their scientific assessment on the synthesis of information performed by internal assessors, from clinical data provided by the manufacturers or retrieved from an in-house scientific literature search. They focus specifically on the following features of studies: methodological quality; selection of a relevant population; main outcome measure; and adverse events and complications related to the procedure. For every assessment, a public document summarising supportive clinical data and providing the opinion is available on the French National Authority for Health’s website [7].

Previous studies have shown that the approval process for high-risk medical devices in the USA is often based on insufficiently robust studies, suggesting that evidence prior to marketing may not be adequate [9,10]. This study aimed to ascertain what level of evidence was available for IMDs access to reimbursement in France. Therefore, we have described the scientific evidence, in terms of the methodology and characteristics of the clinical studies used by CNEDIMTS for implantable medical device assessment, after marketing but prior to the decision on reimbursement.

## Methods

### Opinions

An opinion is the result of CNEDIMTS assessment of safety and efficacy of a device in a specified indication, expressed by the qualification of its “expected benefit” (EB) as insufficient or sufficient; in the latter case its “improvement of expected benefit” (IEB) level is documented. EB is a multifactor criterion based on expertise of clinical benefit, efficacy/safety ratio, and public health benefit. IEB is given in comparison to the established standard of care: level I means there is a major improvement in expected benefit with the IMD; level II is a significant improvement; level III is a moderate improvement; level IV is a minor improvement; and level V is no improvement. An IMD could be used in different indications, resulting in one opinion for each indication. Therefore there could be more opinions than devices; the statistical unit for this study was one opinion, given for one IMD used for one precise clinical indication.

Opinions can be issued for a new application (first or subsequent), a modification, or a renewal. A “new application” was considered as “first”, only for the first application, and as “subsequent” when re-application was due to a previous insufficient EB opinion. After the IMD is included on the list, each modification (e.g. restriction of use or indication) must be assessed by CNEDIMTS. Every 5 years (maximum) after inclusion on the list, a renewal application must be submitted, leading to assess the observed benefit and the improvement of observed benefit. For convenience, EB will refer to both expected (new application) and observed (renewal application) benefit.

Public documents presenting opinions summarise information on clinical data: the number of studies provided by the applicant, the number of studies selected by CNEDIMTS for the assessment process, and the abstract outlining the main characteristics of selected studies (design, number of patients, results). Supporting clinical data are specific or non-specific to the device assessed. Non-specific data relating to the device come from studies performed on an equivalent medical device (same technological characteristics and same intended use) i.e. previous model or device manufactured by a competitor.

### Search and opinion selection

The present study included all opinions concerning IMDs issued by the CNEDIMTS in 2008 and publicly available on the website of the French National Authority for Health ( http://www.has-sante.fr).

### Data collection

For each opinion, two reviewers (LH and ED) independently examined and collected the data using predefined case report forms. Discrepancies between the two reviewers were resolved by consensus.

#### Study selection within opinions

Only studies reporting efficacy were examined. For modification or renewal applications, only the new clinical data, i.e. that became available since the previous opinion, were considered. The reviewers extracted the number of studies: i) provided by the manufacturer (when explicitly provided); and ii) selected by CNEDIMTS (including those from manufacturer and those from the in-house literature search) as mentioned in its opinion.

Among the studies selected by CNEDIMTS, the one presenting the highest level of evidence was chosen for methodological description, whether it was specific or non-specific to the device. The levels of evidence of the studies were defined as follows (from the highest to the lowest [11]): 1) meta-analysis of randomised controlled trials (RCTs); 2) RCTs; 3) non-randomised comparative studies; 4) non-comparative studies i.e. meta-analysis of non-comparative studies, prospective observational registries, prospective case-series, and retrospective case-series. Systematic literature reviews were considered if no specific study was available. When two studies had the same level of evidence, the one with the highest number of patients was analysed.

#### Data items

The following general information was extracted: the date of the opinion; the year of the CE marking (*Conformité Européenne*); the type of application i.e. new application (first or subsequent), modification, or renewal; the date of previous opinion if relevant; and the opinion result i.e. EB qualification (sufficient or insufficient) and IEB level.

The following main methodological characteristics of the highest level of evidence studies, as available in public documents, were collected:

– number of centres (single centre, national multicentre, and international multicentre);

– study design (as described above);

– total number of patients included in statistical analysis;

– output (statistical significance of the main result).

When no studies had been retained as scientifically sound by the CNEDIMTS, we collected the other sources of data (if any) used to provide the opinion.

### Data analysis

The categorical variables were described as frequencies and percentages, and the continuous variables as average or median and range (minimum and maximum). For analysis, we considered a variable “EB + IEB” defined with 6 categories: insufficient EB, IEB level V, IEB level IV, IEB level III, IEB level II, and IEB level I. Types of application and collected studies characteristics were cross-tabulated with the results of the opinions (EB + IEB), and types of application were cross-tabulated with study design. Fisher exact tests were used. All data analyses were performed using SAS version 9.2 (SAS Institute Inc, Cary, NC).

## Results

### Description of opinions

**Figure 1:**
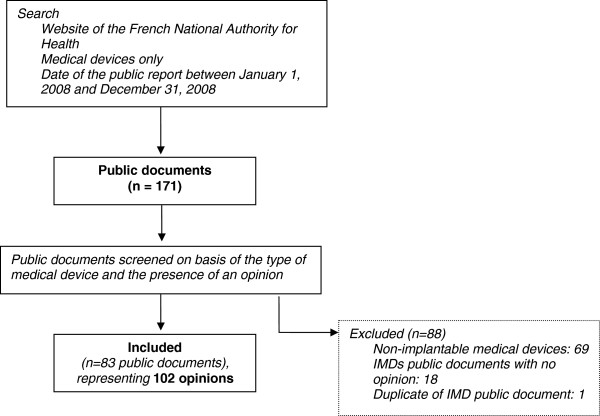
Flow diagram of opinion selection.

A total of 171 public documents were issued by the CNEDIMTS for medical devices in 2008; 83 concerning 93 IMDs were included in the analysis (Figure 1), representing 102 opinions.

Seventy-nine opinions (77.5%) resulted in sufficient EB (Table 1). One IEB level I was observed (deep brain stimulation medical device).

**Table 1:** Distribution of the Expected Benefit and Improvement of Expected Benefit level according to the type of application

	**Total N (%)**	**New N (%)**	**Modification N (%)**	**Renewal N (%)**
Sufficient EB*	79 (77.5)	46 (68.7)	13 (92.9)	20 (95.2)
*IEB*^*†*^*level I*	*1 (1.0)*	*0 (0.0)*	*1 (7.1)*	*0 (0.0)*
*IEB level II*	*8 (7.8)*	*2 (3.0)*	*0 (0.0)*	*6 (28.6)*
*IEB level III*	*5 (4.9)*	*3 (4.5)*	*1 (7.1)*	*1 (4.8)*
*IEB level IV*	*15 (14.7)*	*11 (16.4)*	*1 (7.1)*	*3 (14.3)*
*IEB level V*	*50 (49.0)*	*30 (44.8)*	*10 (71.4)*	*10 (47.6)*
Insufficient EB	23 (22.5)	21 (31.3)	1 (7.1)	1 (4.8)
Total	102	67	14	21

* EB: expected benefit.

^†^ IEB: improvement of expected benefit.

Sixty-six percent of the opinions resulted from new applications (67/102). The opinions were issued a median of 5.0 years after the CE marking for first new applications (range: 0.0 to 12.0). Concerning subsequent new applications, the new opinion was given a median of 4.4 years after the previous application (range: 2.9 to 6.1). Opinions concerning renewals were given after a median of 3.0 years following the previous one (range: 1.5 to 6.0).

EB + IEB differed according to the type of application (p = 0.002). Of the 23 opinions with insufficient EB, 21 were new applications (91.3%).

### Description of clinical data

For 70/102 opinions (68.6%), at least one clinical study was provided by the applicant, with an average number of studies provided of 3.4 in these cases (the precise number of studies provided was available for 54 opinions; median: 2, range: 1 to 15). The CNEDIMTS analysed clinical studies in 72/102 opinions (70.6%), with an average of 3.2 selected and analysed studies (median: 2, range: 1 to 15).

Among these, the study with the highest level of evidence concerned the relevant medical device in 45/72 studies (62.5%), a previous model 15/72 (20.8%) and a similar model in 12/72 (16.7%).

The main methodological characteristics of these studies are shown in Table 2.

**Table 2:** Description of methodological characteristics of the highest level of evidence studies considered for assessment of implantable medical devices (one study analysed per opinion)

	**Overall N (%)**	Sufficient Expected Benefit (EB*)					**Insufficient EB**
		IEB* I	**IEB II**	**IEB III**	**IEB IV**	**IEB V**	
**Number of centres**^†^							
Single centre	12 (19.7)	0	0	1	2	4	5
National multicentre	16 (26.2)	0	1	0	4	8	3
International multicentre	12 (19.7)	1	0	2	3	1	5
Not known	21 (34.4)	0	2	1	5	9	4
**Design**							
Meta-analysis of RCTs^‡^	5 (6.9)	0	5	0	0	0	0
RCTs	25 (34.7)	1	3	3	7	7	4
Comparative non-randomised study	4 (5.6)	0	0	0	0	1	3
Non-comparative study	34 (47.2)	0	0	1	8	15	10
*Retrospective case series*	*5 (14.7)*	*0*	*0*	*0*	*0*	*2*	*3*
*Prospective cohort*	*21 (61.8)*	*0*	*0*	*1*	*5*	*9*	*6*
*Observational registry*	*6 (17.6)*	*0*	*0*	*0*	*2*	*3*	*1*
*Meta-analysis of non comparative study*	*2 (5.9)*	*0*	*0*	*0*	*1*	*1*	*0*
Systematic literature review	4 (5.6)	0	0	1	0	1	2
**Number of patients**^§^							
(median (range))	127.5 (8-18 023)	40 (.)	530 (22-18 023)	568.5 (8-1 065)	67 (15-859)	198 (29-8 318)	62 (19-280)
**Output**^||^							
Significant result	21 (70.0)	1	8	2	3	4	3
Insignificant result	9 (30.0)	0	0	1	1	4	3
***Total***	***72 (100.0)***	***1***	***8***	***5***	***15***	***24***	***19***

* EB: expected benefit; IEB: improvement in expected benefit.

^†^ Non-applicable for 11 studies (meta-analysis and systematic literature reviews).

^‡^ RCTs: randomised controlled trials.

^§^ Non-applicable for systematic literature reviews.

^||^ For comparative studies and meta-analyses of RCTs only; for 4 the information is missing.

The study design was significantly different according to EB + IEB (p < 0.001). The most frequent design was non-comparative studies for insufficient EB (10/19; 52.6%), IEB level V (15/24; 62.5%) and IEB level IV (8/15; 53.3%); randomised controlled trials for IEB level III (3/5; 60.0%) and IEB level I (1/1); and meta-analysis for IEB level II (5/8; 62.5%).

The study design was significantly different according to the type of application (p = 0.006), e.g., for the 45 new applications, there were 24 non-comparative studies (53.3%) compared with 5 for the 18 renewals (27.8%).

For the 30 opinions with no supporting clinical study, 16 (53.3%) were based on CNEDIMTS expert-based analysis (involving multidisciplinary working group using medical device group information: literature analysis and/or previous opinions): 12 concerned implantable cardiac defibrillators; three prosthetic meshes for hernia repair; and one a triple-chamber pacemaker. All concluded with IEB level V. Nine opinions (30.0%) were based on the conclusions of a previous opinion (all concluded with IEB level V). The last 5 opinions reported no data and concluded with insufficient EB for 4, and IEB level V for 1.

## Discussion

In 2008, among the clinical studies with the highest level of evidence assessed by CNEDIMTS, less than half were RCTs or meta-analysis of RCTs. The EB for the CNEDIMTS’ opinions on IMDs correlated to the clinical studies’ level of evidence: low levels of evidence were observed in insufficient EB or low levels of IEB. When no clinical data was available for assessment, the opinion was more likely to conclude on insufficient EB.

To our knowledge, this study is the first to provide a description of the methodological characteristics of studies used during medical device assessment by the French National Authority for Health, and one of the first assessments of the decisions issued by a national competent authority. Its main limitation is the use of publicly available opinions as a data source: some data may have been omitted from public documents, as the opinions do not reveal the complexity of the committee’s decision-making process.

The level of evidence of the clinical studies carried out is expected to impact coverage and reimbursement determinations, which indirectly impacts the diffusion of implantable medical devices in healthcare practices. This issue is complicated for manufacturers, who argue that increasing the level of evidence required will create a financial and time barrier to putting new products on the market and restrict patients’ access to new medical technologies [12,13]. This is even more difficult for smaller firms, which represent most of medical technology firms, which may not have the capital to conduct trials and are not equipped to meet regulatory and methodological requirements [14].

In Europe, studies assessing the clinical efficacy of implantable medical devices were not systematically required for CE marking until the 2007 European Directive [15]. The regulatory approval for CE marking focuses on safety, device quality and performance. Very often only scarce information is available when a new medical device first comes onto the market. Indeed, methodological issues are encountered when designing a clinical study for medical devices evaluation [16]. RCTs, commonly used in drug development and considered to be the study design which provides the highest level of evidence for assessing the efficacy of health products, are particularly prone to methodological challenges when considering IMDs [17]. Blinding is not always feasible (or unethical, for instance sham surgery). Interaction between device and operator might induce heterogeneity in clinical efficacy and safety, such as learning curve effect, teams’ expertise and habits (need for procedure standardisation), or continuous technological evolution. Inclusions in such studies might be difficult, since target populations are often small.

In the USA, the safety and effectiveness of high-risk medical devices, such as implantable medical devices, are assessed by the FDA during the Premarket Approval (PMA) process which relies on clinical data. Two studies have shown that PMA for cardiovascular devices between 2000 and 2007 was often based on studies which were insufficiently robust and possibly prone to bias, as only 27% of studies used to support PMA were randomised [9,10]. Furthermore, 65% of PMAs were based on a single study, suggesting that there may not be adequate evidence prior to marketing [9]. Even if most Class III devices should require PMA, it has been discussed that a large percentage avoid this process and go through 510(k) submission, which is known to be less rigorous with no systematic need for supportive clinical evidence [18]. In 2003-2007 fiscal years, 228 Class III devices had been cleared by FDA through 510(k) process, while 217 had been submitted through original PMA [19].

We showed that a new opinion was given up to 12 years after CE marking. During this time, a new device may be distributed and widely used by physicians in their standard practice of care, especially in the case of innovative devices [20]. Once the medical device has been distributed, it becomes difficult to assess its clinical benefit and cost in relation to existing strategies [21]. The main constraint in performing RCTs after device distribution is that randomisation may not be accepted, not only by physicians, but also by patients who are well informed about new technologies and are keen to access them. Therefore, to determine efficacy and safety, alternative approaches to conventional trial designs might be considered, e.g. use of historical controls and acceptance of p values greater than 0.05 [22]. It has also been suggested that well-designed comparative observational studies may provide information for clinical and effectiveness assessments [23-25]. Such studies may be performed within a post-marketing surveillance program. In first place, post-marketing surveillance has been set up to explore safety concerns such as rare adverse events, through vigilance reporting. However, specific post-marketing clinical studies have to be developed as they can offer the opportunity to collect outcomes data [26], not only for safety but for efficacy purpose. It has been suggested for high-risk medical devices that a post-marketing surveillance program should be in place at the time of market authorisation as a continuum of clinical studies from pre-market to routine use, within a new regulatory approach [27]. A new model based on temporary authorisations and post-marketing studies could be developed. Currently, post market registries are being increasingly set up to provide real life data about use, safety and efficacy of IMDs.

HTA for medical devices can guide collective choices in terms of access to technology, with a view to ensuring their effectiveness and cost-effectiveness. The lack of scientific data raises ethical issues as offering a new, unapproved technique outside of a clinical research context may put the patient at risk. The timely diffusion of such techniques is vital as there are risks associated with the premature introduction of a device without sufficient clinical evaluation, just as an excessive waiting period may be detrimental to patients. There is a serious question as to when the assessment should be carried out: if it is done early in the development process, both the device and procedure may undergo changes and the learning curve effect has to be incorporated, and the findings of the assessment may rapidly become obsolete [28]. Procedures to ensure the “safe” diffusion of innovations has been implemented in European countries as programmes for funding innovation under the framework of clinical research. A programme to support innovative and costly technologies was created in the early 2000s by the French Ministry of Health, with the aim of supervising their diffusion whilst simultaneously assessing their clinical and cost effectiveness. In the United Kingdom, a procedure for temporary reimbursement has been set up for innovative devices when used only in research for the purposes of generating clinical data and good practice guidelines [29].

## Conclusions

This study confirmed that level of evidence of clinical evaluation of IMDs is low and needs to be improved, since less than half of clinical studies with the highest level of evidence assessed by CNEDIMTS in 2008 were RCTs or meta-analysis of RCTs. Future work should design new recommendations in this field by investigating the determinants and the solutions needed to improve quality of clinical evaluation of medical devices, in connection with small and medium firms, clinicians and authorities.

## Abbreviations

CNEDIMTS: Commission Nationale d’Evaluation des Dispositifs Médicaux et Technologies de Santé; EB: Expected benefit; FDA: Food and Drug Administration; HAS: Haute Autorité de Santé; HTA: Health technology assessment; IEB: Improvement in expected benefit; IMD: Implantable Medical Device; PMA: Premarketing authorisation; RCT: Randomized controlled trial.

## Competing interests

The authors declare that they have no competing interests.

## Authors’ contributions

LH designed the study, examined, collected and managed the data, interpreted data and drafted the manuscript. ED participated in the design of the study, examined and collected the data, did the statistical analysis, and helped to draft the manuscript. KMB participated in the design of the study and the collection of data. FC participated in the design of the study, and helped to draft the manuscript. All of the authors read and approved the final manuscript.

## Pre-publication history

The pre-publication history for this paper can be accessed here:

http://www.biomedcentral.com/1471-2458/12/585/prepub
